# The Harvard organic photovoltaic dataset

**DOI:** 10.1038/sdata.2016.86

**Published:** 2016-09-27

**Authors:** Steven A. Lopez, Edward O. Pyzer-Knapp, Gregor N. Simm, Trevor Lutzow, Kewei Li, Laszlo R. Seress, Johannes Hachmann, Alán Aspuru-Guzik

**Affiliations:** 1 Department of Chemistry and Chemical Biology, Harvard University, Cambridge, Massachusetts 02138, USA; 2 Department of Chemical and Biological Engineering, University at Buffalo, The State University of New York, Buffalo, New York 14260, USA; 3 Computational and Data-Enabled Science and Engineering Graduate Program, University at Buffalo, The State University of New York, Buffalo, New York 14260, USA; 4 New York State Center of Excellence in Materials Informatics, Buffalo, New York 14203, USA

**Keywords:** Energy, Solar cells, Electronic structure

## Abstract

The Harvard Organic Photovoltaic Dataset (HOPV15) presented in this work is a collation of experimental photovoltaic data from the literature, and corresponding quantum-chemical calculations performed over a range of conformers, each with quantum chemical results using a variety of density functionals and basis sets. It is anticipated that this dataset will be of use in both relating electronic structure calculations to experimental observations through the generation of calibration schemes, as well as for the creation of new semi-empirical methods and the benchmarking of current and future model chemistries for organic electronic applications.

## Background & Summary

Standard data sets used for the calibration of computational results have been extremely useful for the development of electronic structure methods and their application to areas such as thermochemistry^1–3^ as well as non-covalent interactions^4,5^. To our knowledge, the field of organic photovoltaics, as it pertains to high-throughput virtual screening^6–8^, lacks a similar collection of data. Since the relationship between theoretically predicted and experimentally observed properties is often non-trivial, the dissemination of directly comparable data for a well-defined set of molecules can be a great asset to accelerate advances in this field.

Many areas of materials chemistry have benefited from the application of high-throughput virtual screening, which has led to an accelerated discovery of new materials^6–16^. Since this approach allows a large number of compounds and materials to be pre-screened using efficient *in silico* (often quantum-chemical) techniques, it allows experimental scientists to focus time and resources on fewer, more promising, candidates^17^. However, theoretical studies (*i.e.*, based on density functional theory (DFT)) only approximates the observed experimental properties and care must be taken when relating one to the other^18^. The Scharber model^19^ is utilized to compute the maximum percent conversion efficiencies for the 350 studied molecules. The quantities that enter the Scharber model are the lowest unoccupied molecular orbital (LUMO) and highest occupied molecular orbital (HOMO) energies energy and the HOMO-LUMO gap. These are used to compute the open circuit potential (*V*_OC_) and short circuit current density (*J*_SC_). Percent conversion efficiency (PCE) is the computed according to equation 1.

(1)PCE=100∗VOC∗FF∗JSSPin

In the Scharber model, the fill factor (FF) is set to 65%, and *J*sc is qualitatively related to the HOMO-LUMO gap.

One area in which this method has been most visibly applied is the area of organic photovoltaic materials^6,7^, with the Harvard Clean Energy project being an example^8,20^. Many approximations are made to efficiently screen of millions of compounds. An ability to relate these calculations to experimental data is critical for the implementation of an efficient feedback loop. We believe that such a feedback loop is vital for the ongoing success of collaborative efforts. Unfortunately, there are very few collections of experimental results from which to build these models, and we are not aware of any significantly sized set of molecules for which both quantum chemical and experimental values are reported.

Here we report the Harvard Organic Photovoltaic Dataset (HOPV15) consisting of both experimental results compiled from the literature, and corresponding data from quantum chemical calculations using a selection of five functionals chosen to contain both generalized-gradient approximation (BP86 (refs 21,22) and hybrid designs with a range of incorporated amounts of exact exchange PBE0 (refs 23,24), B3LYP^21,25^, and M06-2X^26,27^ in combination with the double-ζ def2-SVP basis set^28^. It will have a multitude of uses, including the calibration of quantum chemical results to experimental observables^29^, the development of new methodologies for property estimation^19^, as well as the design of new Hamiltonians for semi-empirical methods^30^.

**Figure 1:**
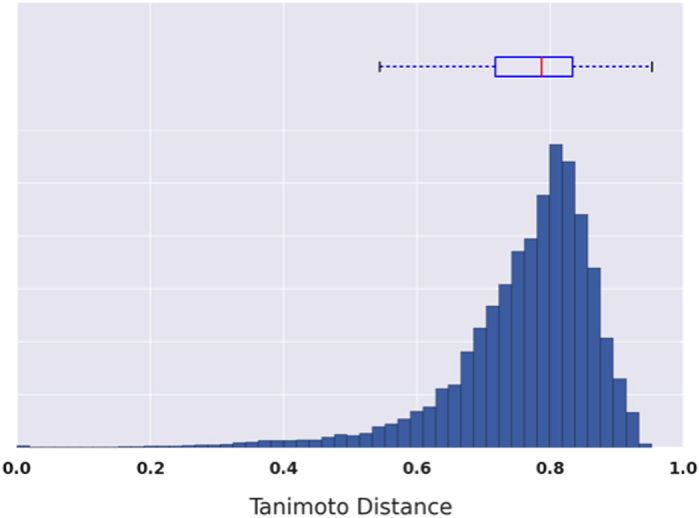
The distribution of Tanimoto distances in the distance matrix calculated for the Harvard Organic Photovoltaic 2015 dataset presented in this work suggests that the data set encapsulates significant molecular diversity. The box-plot above the histogram shows the mean, 25 and 75% percentile values with 10 and 90% points indicated by the whiskers.

The compounds in this data set represent a diverse cross-section of molecular designs in this field. This is reflected in the Tanimoto distance between each molecule and all others as described by the 512-bit, radius-2 Morgan circular fingerprint^31^. We only calculate the upper triangular of the distance matrix. A histogram of the computed distances is shown in Fig. 1 and it emphasizes that the average Tanimoto distance is just below 0.8 (the Tanimoto distance is bounded at 0 for a perfect match between fingerprints and 1.0 for no common bits in the fingerprint). An average distance of 0.8, therefore, is a good indication of the molecular diversity of this data set.

## Methods

### Simplification of the Conformational Energy Landscape

Many of the structures reported in the literature had long alkyl chains added to the ‘active’ photovoltaic core in order to improve solution processing. This substantially convolutes the conformational energy landscape, while the electronic structure are not significantly changed. This was confirmed by studying the effect of chain length on the HOMO-LUMO gap of these molecules with a) the original chain length, b) the chain reduced to two carbons c) the chain length reduced to one carbon and d) the chain removed entirely. It was observed that there was no significant difference on the HOMO-LUMO gap when the chain was reduced to one or two carbons (ΔGap=0.0±0.01 eV), and a small difference when the chain was removed altogether (ΔGap=0.05±0.08 eV). Since a complicated conformational landscape necessitates the generation of an exponentially growing number of conformers (the number of conformers scales approximately as 3^*N*^ where *N* is the number of rotatable bonds) and can thus reduce the performance of many of the common conformer generation algorithms, we decided to truncate alkyl chains to a methyl group.

### Generation of Molecular Conformations

Starting from the simplified molecular-input line-entry system (SMILES^32^) string representation of the molecule, with all alkyl chains reduced to one carbon, 1500 initial guesses at the 3D conformation of the molecule were generated using the conformer generation package included in the open-source RDKit software^33^. These initial guesses were then minimized using the MMFF force field^34^ implemented in this package^35^, with duplicate structures resulting from initial guesses minimizing to the same relaxed structure removed using the *obfit* functionality implemented in the *OpenBabel* software package^36^. The lowest energy conformation from each of up to twenty clusters which fell within a window of 5 kcal mol^−1^ were selected to represent energetically feasible conformations for the molecule which may contribute to the performance of the material, especially in disordered or semi-ordered materials.

### Quantum-chemical Calculations

The geometries for every selected conformation were minimized using the BP86 functional, and the def3-SVP basis set. For force-field minimizations, duplicate structures resulting from multiple force field minima converging to the same BP86/def2-SVP minimum were removed using the *obfit* functionality implemented in OpenBabel software package^36^ with a tolerated RMSD in atomic positions of >0.1 Å.

For each unique conformation, single point energies were calculated with PBE0 (refs 23,24), B3LYP^21,25^, and M06-2X^26,27^ in combination with the double-ζ def2-SVP basis set^28^. As previously stated, these functionals represent a range of exact exchange, with BP86 (refs 21,22) (0%) and M06-2X^26,27^ (52%) representing the extremes of the range. The inclusion of a range of functionals increases the utility of the data set since the additional information can be used to either benchmark performance against a range of model chemistries, or alternatively these results can be used in an *ensemble average* to provide a model, which is more general than any individual model chemistry.

### Code availability

Quantum-chemical calculations were performed using Q-Chem version 4.1.2, and is available from http://www.q-chem.com under a commercial license.

The OpenBabel software package is freely available from http://openbabel.org/ under the GPL license^37^.

The RDKit is freely available from http://rdkit.org/ under the BSD licence^38^.

## Data Records

The data set is shared publically on *Figshare* (Data Citation 1). We extensively searched the literature and located 350 small molecules and polymers that were utilized as *p*-type materials in OPVs. For each reported molecule, atomic coordinates, experimental properties and their calculated equivalents are stored in a plain-text XYZ-format described below. Deposited are the 350 molecules which make up the HOPV dataset, up to twenty of their low-energy calculated molecule conformations and, where available, the power conversion efficiency (PCE), open circuit potential (*V*_OC_), short circuit current density (*J*_SC_), highest occupied molecular orbital (HOMO) energy, lowest unoccupied molecular orbital (LUMO) energy and the HOMO-LUMO gap. The reported PCE, *V*_OC_, *J*_SC_, HOMOs, and LUMOs are reported in percent, Volts, mA/cm^2^, and atomic units, respectively.

### File format

**Figure 2:**
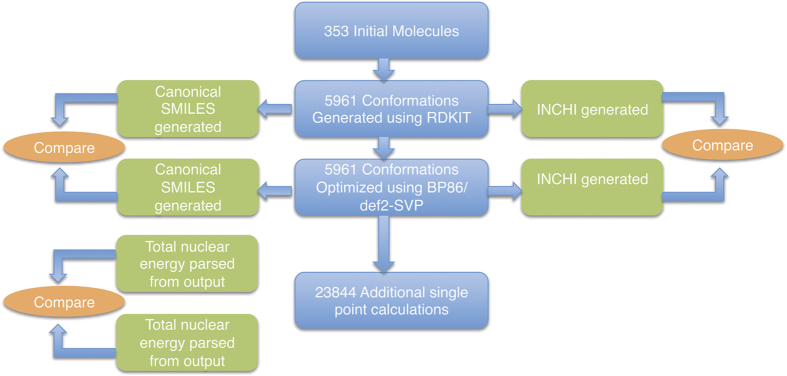
The workflow for validating calculated geometries by comparing the canonical SMILES representation and the InChI representation generated for the initial force field optimized geometry for a conformer and the DFT optimized geometry for that conformer. The total nuclear energy for each conformer was calculated and compared to that reported in the related quantum-chemical output files.

Figure 2 shows the makeup of the HOPV_15.data file (per molecule). It is an extension of the commonly used XYZ format for encoding Cartesian coordinates of molecules, with no formal specification. It contains a header line specifying the number of atoms n, a comment line, and n lines containing element type and atomic coordinates, one atom per line. We have extended this format as indicated in Table 1 in a manner similar to Von Lilienfeld *et al.*^39^ did for purposes of machine learning. In addition to the XYZ format for the storing of electronic coordinates, experimental and calculated properties are stored in CSV format as described in Tables 2 and 3, respectively.

## Technical Validation

### Validation of Computational Results

For each conformer generated from the initial molecules, a DFT optimized geometry was generated at the BP86 (refs 21,22)/def2-SVP^28^ level of theory. Geometries were validated using a technique similar to that used in the work of von Lilienfeld *et al.* ^40^ To detect instances where the DFT optimized geometry had changed drastically from the initial force field optimized geometry, both the InChI and canonical SMILES were generated for both geometries, and compared. The InChI and canonical SMILES are both theoretically unique identifiers, and so comparing these two descriptors represents a method for evaluating if the minimized structure consistent with typical geometries.

In order to validate the computation of the electronic structure of the conformations, an additional test was performed on all optimizations, and single point electronic structure calculations. The total nuclear energy, a property, which is solely reliant on the nuclear positions and charges, was calculated for each conformation. This was then compared to the reported values within the Q-Chem^41^ output files for each calculation. This technique has been utilized as part of the Harvard Clean Energy Project’s validation suite for calculations performed on the World Community Grid^42^ and is aimed at testing for hardware issues which may negatively influence the quality of the calculated result. All calculations on molecules within the HOPV15 dataset passed both of these tests, which demonstrates the validity of the data set.

### Validation of Experimental Results

The experimental results contained within this data set are taken from the literature, and so have been validated using the peer-review system. Wherever possible, molecules were taken from reviews, and cross referenced against the original publication to reduce the potential for transcription errors. In this way, the choice of molecules and the quality of the data has been validated by an external scientist (the composer of the review) who is also a domain expert. Where multiple reports for the same architecture exist, the most recent value was taken.

## Additional Information

**How to cite**: Lopez, S. A. *et al.* The Harvard organic photovoltaic dataset *Sci. Data* 3:160086 doi: 10.1038/sdata.2016.86 (2016).

## Supplementary Material



Supplementary information

## Figures and Tables

**Table 1:** A description of the file-format used in the HOPV15 data file.

**Line**	**Contents**
1	SMILES of molecule
2	InChI of molecule
3	Experimental data (as CSV)
4	‘Pruned’ smiles of molecule
5	Total number of conformers
	For each conformer (N=index, n= number of atoms)
L=5+(N *n)	Conformer number
L+1	Number of atoms
L2=L+2-> L+2+(N*n)	Atomic element, X, Y, Z coordinates
	For each functional:
L2+1-> L2+5	Calculated Data (as CSV)

**Table 2:** A description of the CSV format for storing experimental information.

1	Digital Object Identifier
2	InChIKEY of molecule
3	Construction (Polymer/molecule)
4	Architecture
5	Complement
6	HOMO
7	LUMO
8	Electochemical gap
9	Optical gap
10	PCE
11	*V*_OC_
12	*J*_SC_
13	Fill factor

**Table 3:** A description of the CSV format used to store calculated properties.

**Index**	**Contents**
1	Functional/Basis set description
2	HOMO
3	LUMO
4	Gap
5	Scharber PCE
6	Scharber *V*_OC_
7	Scharber *J*_SC_
